# Clinical evaluation of the function of hypothalamo-pituitary-thyroid axis in children with central nervous system infections

**DOI:** 10.1186/1824-7288-37-11

**Published:** 2011-02-11

**Authors:** Fuyong Jiao, Xiaoyan Zhang, Taomin Bai, Jing Lin, Wei Cui, Bingweng Liu

**Affiliations:** 1 Department of Pediatrics, Shaanxi Provincial People's Hospital, Xi, an, China; 2 Department of Pediatrics, The Second Teaching Hospital, Yanan Medical College, Suide, China; 3 Department of nuclear medicine, The Second Teaching Hospital, Yanan Medical College, Suide, China

## Abstract

**Background:**

It is well known that certain non-thyroidal critical illness may lead to euthyroid sick syndrome(ESS). There are little reports about the change of thyroid hormone in the children's central nervous system (CNS) infections.

**Results:**

The results of serum TT3, TT4 and TSH in these children were compared with those in 20 cases of healthy adults and 20 cases of adults with primary hypothyroidism. Serum T3 and T4 were decreased in 34/78 children with CNS infections, T3 and T4 were much lower than those of healthy adult (p < 0.05), but still higher than that of the primary hypothyroidism (p < 0.05), and TSH levels were not significant differences among children with CNS infections and children with non-CNS infections (p > 0.05).

Low T3 and T4 levels in serum and cerebrospinal fluid(CSF)were predominant in children with serious infections of CNS, 31/34 (percent 91.17) cases of serious CNS infection had low serum TT3 and/or TT4. The low T3 with low T4 was seen in 14/34 children of severe CNS infections, 3 of them died. The levels of CSF T3 (X ± SD = 0.39 ± 0.47 ng/ml) and T4 (x ± SD = 1.02 ± 1.27 ug/dl) in the serious CNS infections were lower than that of non-CNS infections T3 (x ± SD = 0.93 ± 1.23 ng/ml), and T4 (x ± SD = 2.42 ± 1.70 ug/dl), 7 died children were all in the subjects of low T3 and/or low T4.

In 22 children with non-CNS infections, serum T3 and T4 levels were lower than that of healthy adult, but have not significant difference(p > 0.05).

**Conclusions:**

These results suggest that detection of TT3, TT4 and TSH in serum and/or CSF simultaneous or alone in analyses would be valuable in correctly judging thyroid function and evaluating the prognosis of the children with infections of CNS. Measuring a little amount of blood (1 ml)or CSF required for this method is a simple, convenient and accurate method.

## Background

Numerous studies have demonstrated a high incidence of hypothyroidism in non-thyroidal illness in general. Anyone who has worked in an intensive care unit is aware of the phenomenon commonly referred to as the nonthyroidal illness syndrome (NTIS) or euthyroid sick syndrome (ESS), observed in approximately 44% of these patients [1]. Abnormalities in thyroid function tests in patients with non-thyroidal illness may be divided into low T3 syndrome, low T3 and low T4 syndrome, high T4 syndrome and a mixed form. Hence it is becoming clear that the conversion of T4 to T3 can vary according to the clinical state, which can therefore influence the metabolic action of thyroid hormone and the function of hypothalamo-pituitary-thyroid axis.

The severity of these neuroendocrine alterations was shown to be related to adverse outcome of patients in the intensive care unit [2]. When thyroxine (T4) falls to < 4 μg/dl, the risk of death rises to ~50%, and when T4 falls to < 2 μg/dl, mortality increases even more to ~80% [3,4].

There are little reports about the change of thyroid hormone in the central nervous system (CNS) infections of children. We have measured total T3 and T4 concentration of serum and/or cerebrospinal fluid (CSF) in children with infections of CNS, in healthy adults and in adults with primary hypothyroidism, children with non-CNS infections respectively, in order to find the changes of thyroid hormone in serum and/or CSF in relation to CNS infections.

## Methods

The study included 145 children of non-thyroidal illnesses, 93 male and 52 female. 123 with CNS infections and 22 with non-CNS infections. Out of the 123 with CNS infections, 39 had purulent meningitis, 7 meningococcal meningitis, 21 tuberculous meningitis and 56 viral encephalitis. The classify of severity of CNS infections was based on the clinical manifestation and laboratory examinations, and electroencephalogram (EEG) examination. The severe CNS infections had these symptoms such as seizure, respiratory failure, conscious disturbance, persistent fever, and obviously abnormality in EEG. Out of 22 children with non-CNS infections, 16 had urinary system diseases, 6 had respiratory system diseases. In addition, we have measured 20 adults of primary hypothyroidism; 20 cases of healthy adult. The Patient characteristics appear in table 1. Samples of serum and CSF were collected from children with non-thyroidal illness in our hospital. The diagnosis was based on the clinical manifestation and laboratory examinations of blood and CSF, and EEG examination. No children had thyroid malformation and history of thyroid diseases.

**Table 1:** subjects characteristics

*Groups*	*Number*	*Age (years)*	*Sex (male/female)*
Purulent meningits	39	6 ± 6.97	24/15
Meningococal meningits	7	7 ± 6	4/3
Tuberculous meningits	21	7.2 ± 6.1	15/6
Viral encephlitis	56	6.8 ± 6.2	34/22
Respiratory system disease	6	7.5 ± 5.6	4/2
Hypothoridism	20	30 ± 12	12/8
Healthy adult	20	32 ± 13	12/8
Urinary system disease	16	8 ± 6	10/6

Serum and/or cerebrospinal fluid (CSF) concentration of total triiodothyronine (TT3) and total thyroxine (TT4) and thyrotropin (TSH) were measured in 145 children of non-thyroidal illness, in which 123 children were CNS infections. We measured serum and CSF T3 and T4 in 36/123 children, serum T3 and T4 in 42/123 children, CSF T3 and T4 in 45/123 children. We also measured CSF T3 and T4 in 6 children with respiratory system diseases. The results of serum T3, T4 and TSH in the children with non-thyroidal illness were compared with those in 20 cases of healthy adults and 20 cases of adults with primary hypothyroidism.

The serum and CSF samples were obtained by venipuncture and lumbar puncture before initial treatment. The specimens were stored at -20°C until analysis. The serum and CSF T3, T4 and TSH were measured by radioimmunoassay (RlA), the serum and CSF T3 and T4 were measured by double-antibody plus PEG, and the serum TSH was measured by double-antibody. Radio-immunoassay kit is manufactured by institute technique of analysis in Shanghai and Sichuan, China.

All samples were measured with double-tubules, then re-measured by Type FJ-2000 R counter radioimmunoassay (made in Xian National 262 factory). The rate of recovery in T3 and T4 were 96 to 108 percent, coefficient of variation (CV) within the batch and CV among the batch in T3 and T4 were 6 to 7 percent and 8 to 9 percent respectively. The rate of recovery of TSH was 97.8 percent, CV within the batch and CV among the batch were 6 percent and 4.8 percent respectively. Each measurement was required to be < 0.04 for reaction error relation (RER). Data were expressed as mean ± standard deviation (X ± SD). Student's T-test was used for statistical comparisons. T'-test was used when variance not rule. P Value of 0.05 or less was considered statistically significant. The normal values of TT3,TT4,TSH are 1.21 ± 0.43 ng/ml, 9.06 ± 3.15 u g/dl, 3.53 ± 0.42 mu/l respectively.

## Results

The level of serum TT3, TT4 and TSH in all subjects appear in table 2. Serum T3 and T4 were decreased in 34/78 children with severe CNS infections, T3 and T4 were much lower than those of healthy adult (p < 0.05), and TSH levels were not significant differences among children with CNS infections and children with non-CNS infections (p > 0.05). In 22 children with non-CNS infections, serum T3 and T4 levels were lower than that of healthy adult, but have not significant difference (p > 0.05). 37/78 children with CNS infections had low T3 and/or low T4 in the serum. Among them, 3l/37 children were severe infections. 14/31 children with low T4 accompanied by low T3, all them were severe patients, 3 of them died.

**Table 2:** The level of serum T_3_,T_4 _and TSH in all subjects

*Groups*	*Number*	*TT_3_(ng/ml) t/p X ± SD*	*TT_4_(ug/dl) t/p X ± SD*	*TSH(mu/l) t/p X ± SD*
CNS infection mild	44	1.01 ± 0.37 NS※	8.90 ± 2.67 NS※	4.20 ± 2.04 NS※
CNS infection severe	34	0.58 ± 0.28 < 0.05※	5.14 ± 3.42 < 0.05※	4.79 ± 2.04 < 0.05※
Hypothoridism	20	0.29 ± 0.13 < 0.01※	2.93 ± 1.51 < 0.01※	9.06 ± 2.76 < 0.01※
Healthy adult	20	1.05 ± 0.36	8.16 ± 1.30	4.74 ± 0.99
Urinary system disease	16	0.87 ± 0.49 NS※	7.88 ± 3.34 NS※	5.40 ± 2.05 NS※
Respiratory system disease	6	0.63 ± 1.12 NS※	6.53 ± 1.07 NS※	4.97 ± 1.83 NS※

※ compared with healthy adult

The CSF T3 and T4 level in 87 children of CNS infections and 6 children of respiratory system diseases appear in table 3. 60/87 children were mild, other (27/87) were severe. The mild were low T3 and low T4 in CSF, than that of non-CNS infections, but had not statistically significant (p > 0.05). The CSF T4 in severe children was much lower than that of non-CNS infections (p < 0.05).

**Table 3:** The level of CSF T_3 _and T_4 _in patients with CNS infections

*Groups*	*Number*	*TT_3_(ng/ml) t/p X ± SD*	*TT_4_(ug/dl) t/p X ± SD*
Respiratory system disease	6	0.93 ± 1.23	2.42 ± 1.70
CNS infection mild	60	0.56 ± 0.40 NS※	3.81 ± 2.88 NS※
CNS infection severe	27	0.39 ± 0.47 NS※	1.02 ± 1.27 < 0.05※

※ compared with non-CNS infections

## Discussion

This study presents evidence of thyroid dysfunction, expressed as a lower TT3 and TT4 in serum and CSF in children with CNS infections. These findings are in keeping with those of other investigators. Three further findings were observed in the 123 children with CNS infections: (1) the mean T3 and T4 in serum and the mean T4 in CSF were significantly lower in children with severe CNS infections than that in healthy adults (P < 0.05); (2) The decrease of CSF T4 was higher than that of T3 in children with severe CNS infections; (3) the children with low T3 showed lower survival rate than those with low T4. The data have showed that T3 and T4 in serum are more decreased in severe infections of CNS than that in the mild.

Our data showed that the total T3 and T4 in CSF were much lower than serum T3 and T4 in children with severe CNS infection. This phenomenon can be explained by the fact that brain capillaries have tight junctions, and in this respect it is unlike microvessels of other organ, the brain may be tentatively regarded as a representative peripheral tissue because plasma albumin, thyroid-hormone-binding prealbumin or binding globulins do not cross the brain capillary bed. Thyroid hormones have a low intrinsic permeability in brain endothelia and only small amounts of hormone cross the brain capillary wall via free diffusion. The levels of CSF T3 and T4 in children with severe CNS infections were lower than those in children with non-CNS infections. Thyroid hormone transport system and endothelial permeability in brain could be impaired when suffer from severe CNS infections, so that T3 and T4 in CSF are much decreased. The results show dynamic variation of total serum and CSF T4 and T3 concentrations in accordance with degree of children's conditions.

The mean T3 and T4 of serum by RIA in children with severe CNS infections was lower than that in the healthy adults. The cause of the low T3 level is that production decreases rather than degradation or clearance increase. In this situation, the organism can rapidly reduce its metabolic rate, while serum T3 decreases and reverse T3 increases, so that gain to energy balance. The decreased formation of T3 is reported to be accompanied by an increased reversed T3 (r-T3) value, caused by a reduction of its metabolic clearance as a result of impaired 5'-deiodinase enzyme activity. With the progression of the severity of CNS infections, there is a tendency for total T4 levels to fall. Although many studies have endeavored to evaluate the test to show that the patient with nonthyroidal illness is euthyroid, the serum thyroid hormone levels are subnormal in some patients with a severe systemic illness.

The most common pattern is a decrease in total and unbound triiodothyronin (T3) with normal levels of thyroid stimulating hormone (TSH) and thyroxin (T4). This is classified as SES type 1 (SES-1) or low-T3 syndrome. Classic SES-1 was found in several studies in children, including after bone marrow transplant [5], in Hodgkin's disease, hepatitis, metabolic acidosis due to diarrhoea or diabetic ketoacidosis and sepsis [6]. The de-ionidation from T4 to T3 via peripheral (hepatic) enzymes (inhibition of 5'-deionidase, a selenoenzyme) which is impaired, leading to a decrease of T3 [7]. In general, the severity of illness is correlated to the severity of SES [8]. Very sick patients may show a dramatic fall in total T3 and T4 levels; this state is called the low-T4 syndrome or SES type 2 (SES-2) and has a poor prognosis [9]. Patients with low or undetectable TSH show increased morbidity and mortality [10,11]. Additionally, the response of TSH to thyroid releasing hormone (TRH) is impaired in SES. It was confirmed by Chinga-Alayo and colleagues that the degree of SES seems to have significant influence on a patient's outcome under various conditions [12]. SES-1 is related to good outcome and mild to moderate illness, SES-2 is related to severe illness and poor outcome.

## Conclusions

Measuring TT3, TT4 and TSH in a little amount of blood (1 ml) or CSF from the children with infections of CNS is simple, convenient and accurate. Our studies have showed that detection of T3, T4 and TSH in serum and/or in CSF would be valuable in correctly judging thyroid function and evaluating the prognosis of the children with infections of CNS.

## Competing interests

The authors declare that they have no competing interests.

## Authors' contributions

FYJ initiated the idea of the study, participated in its design, performed the statistical analysis of the results, participated in the coordination and drafted the manuscript. He is the corresponding author of the paper. XYZ performed the statistical analysis of the results, participated in the coordination and helped to draft the manuscript. TMB participated in the sequence alignment and helped to draft the manuscript. All authors have read and approved the final version of the manuscript.
